# Cutaneous Absidia corymbifera in a Lupus Nephritis Patient

**DOI:** 10.7759/cureus.19512

**Published:** 2021-11-12

**Authors:** Kuruvilla K Sebastian, Husam Alzayer, Elizabeth Abraham, Darren Roche, Donal Reddan, David Lappin

**Affiliations:** 1 Department of Renal Medicine, Cork University Hospital, Cork, IRL; 2 Internal Medicine, Mater Private Network, Cork, IRL; 3 Department of Dermatology, University Hospital Galway, Galway, IRL; 4 Department of Nephrology, University Hospital Galway, Galway, IRL

**Keywords:** cutaneous mucormycosis, isavuconazole, lupus nephritis, absidia corymbifera, mucormycosis

## Abstract

A 28-year-old farmer with class IV lupus nephritis presented with a two-week history of a right shin lesion. The lesion was purple in color, fungating, and indurated with a focus of deep ulceration at the inferior pole and punctate, bleeding from its surface. Three months earlier, he was started on induction immunosuppression for a relapse of his lupus nephritis. Since the diagnosis of lupus nephritis, nine years previously, he had had six flares of his disease and had been treated at different time points with cyclophosphamide, rituximab, and high-dose corticosteroids, without adverse events. Laboratory investigations showed improving kidney function (chronic kidney disease [CKD] stage IV) with reducing proteinuria, on his current immunosuppressive regimen. The differential diagnosis for this lesion was calciphylaxis, pyoderma gangrenosum, vasculitic lesion, or an infection. Histology and microbiological analysis confirmed the presence of *Absidia corymbifera.* He was treated with a combination of isavuconazole, reduction of his immunosuppressive agents, excision of the lesion, and skin grafting.

## Introduction

We present in this report a case of cutaneous mucormycosis with a rare opportunistic pathogen, *Absidia corymbifera*, in an immunosuppressed patient with class IV lupus nephritis. Our aim is to describe the natural history of its presentation, the diagnostic process and to finally discuss the complexity involved in treating this fungal infection. We also discuss the challenges faced as a consequence of immunosuppression and the side effects of the treatment in this case report.

## Case presentation

**Figure 1:**
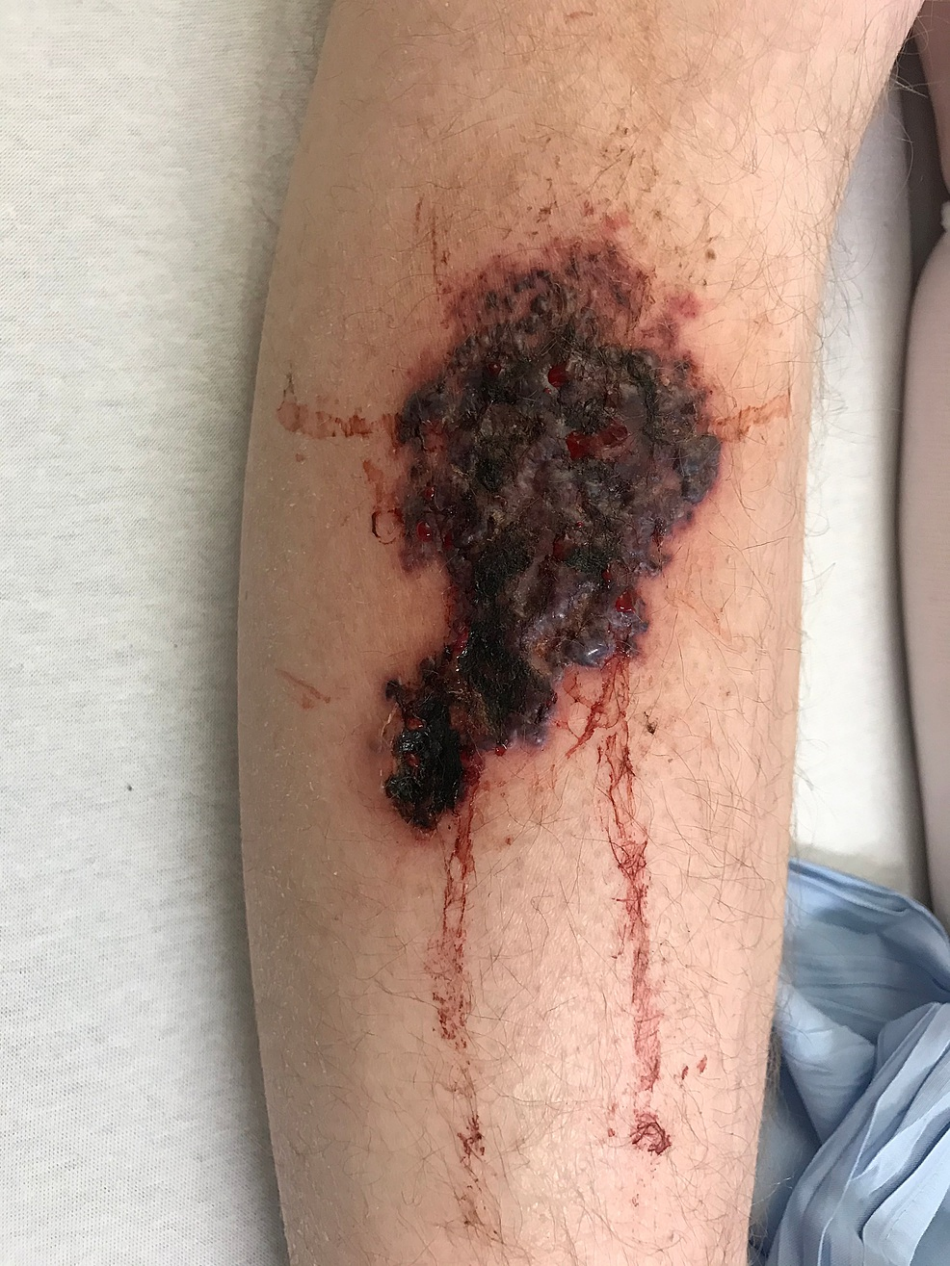
Mucormycosis lesion on the right shin.

A 28-year-old farmer presented with a two-week history of a right shin lesion. The lesion was purple in color, fungating, and indurated with a focus of deep ulceration at the inferior pole and punctate bleeding from its surface (Figure 1).

He had been diagnosed with class IV lupus nephritis nine years ago. He had had six flares of his disease and had been treated at different time points with cyclophosphamide, rituximab, and high-dose corticosteroids, without adverse events. Three months earlier, he was started on Mycophenolate mofetil (one gram twice daily) and high-dose corticosteroids (60 mg daily) for his latest flare of lupus nephritis. His flare was characterized by a rise in creatinine from a baseline of 120 umol/L to a peak creatinine of 550 umol/L and his urine protein creatinine ratio (uPCR) was 433 mg/mmol.

Initial laboratory investigations showed baseline kidney function (chronic kidney disease [CKD] stage IV) with reducing proteinuria on his current immunosuppressive regimen. Inflammatory markers were within normal limits, with no evidence of ongoing lupus activity. A plain X-ray of his right tibia/fibula did not show any abnormality, while an MRI reported soft-tissue edema. A biopsy was performed for further histopathological and microbiological analysis.

**Figure 2:**
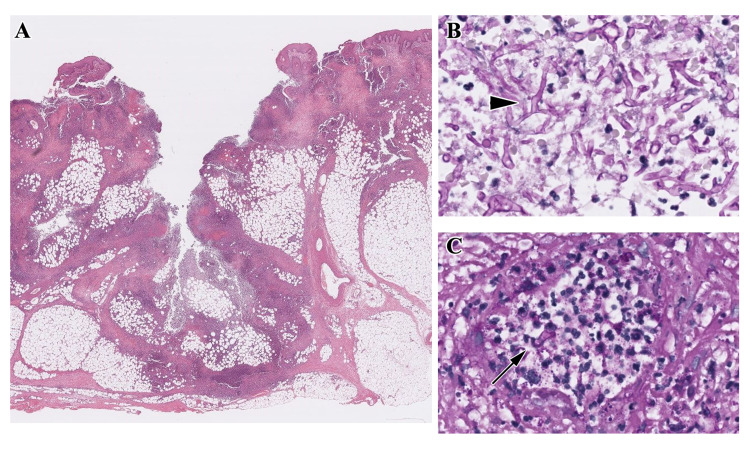
Panel A: Inflammation of the dermis, Panel B: Periodic acid-Schiff (PAS) stain showing fungal hyphae (arrow-head), and Panel C: angioinvasion by fungal hyphae.

Histology of the lesion demonstrated florid acute and chronic inflammatory processes (Figure 2, Panel A), periodic acid-Schiff (PAS) stain for fungal organisms was positive for broad-based fungal hyphae with branching (Figure 2, Panel B, arrowhead) and angioinvasion (Figure 2, Panel C, long arrow). Microbiology confirmed the presence of Absidia corymbifera in the culture of the tissue sample as well as in the ulcer slough.

Upon diagnosis, the lesion was excised, followed by skin grafting. He was initially treated with IV liposomal amphotericin (3 mg/kg once daily) and a reduction of his immunosuppressive agents. Following a deterioration in his renal function, amphotericin was switched to isavuconazole (200 mg IV every eight hours for two days, then 200 mg once daily orally).

**Figure 3:**
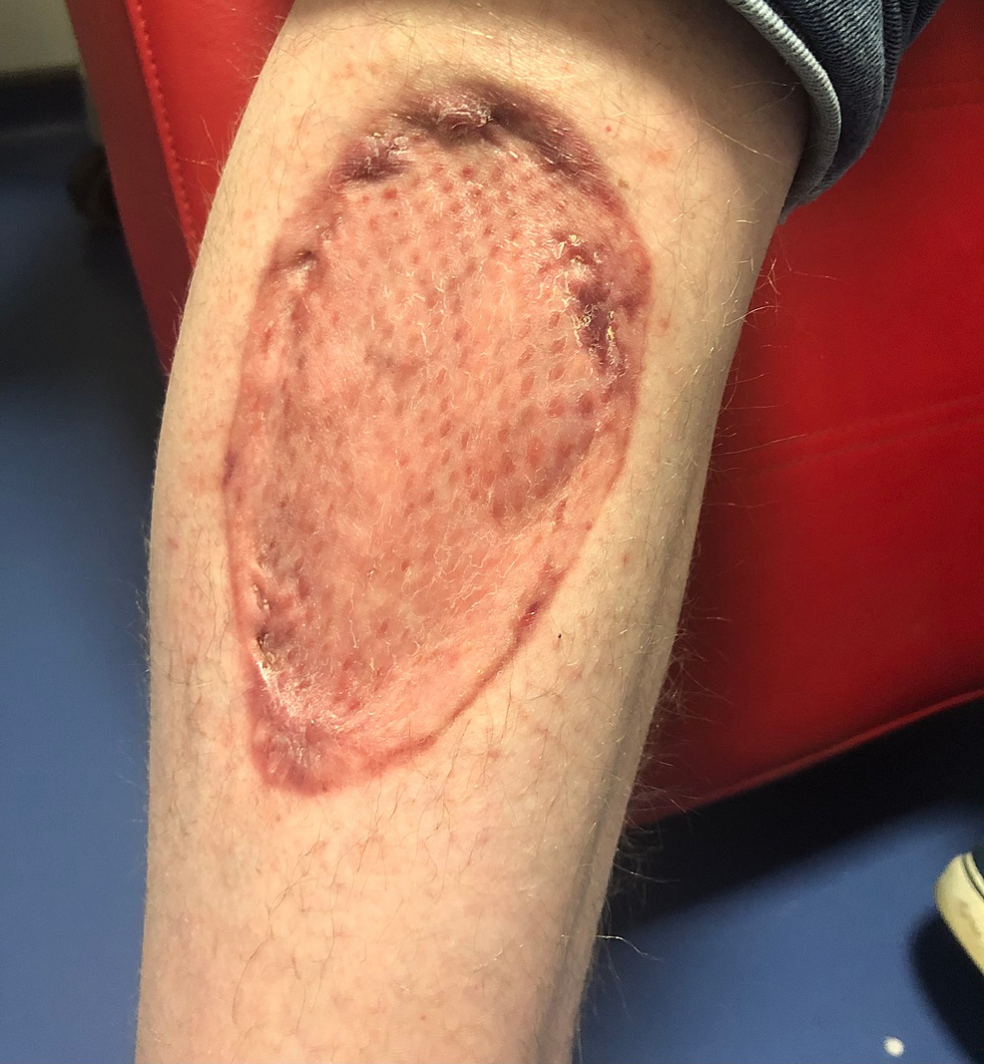
Right shin, three months after lesion debridement and skin grafting.

His treatment was complicated by severe symptomatic anemia, which we believed was due to a combination of myelotoxicity from mycophenolate mofetil and the intravenous preparation of Isavuconazole causing pancytopenia. This particular complication was managed by reducing the mycophenolate mofetil dose and switching IV isavuconazole to an oral preparation and titrating the erythropoietin dosage. The patient also developed hypogammaglobulinemia and was treated with IV immunoglobulin for an interim. He successfully completed three months of treatment with regular monitoring. Figure 3 shows the site of the debrided lesion, three months after skin grafting.

## Discussion

Absidia corymbifera is a rare opportunistic fungus, known to cause pulmonary, CNS, rhinocerebral and cutaneous mucormycosis. Mucormycosis is an infection typically caused by fungi of the class Zygomycetes and of the order Mucorales [1]. The most common species to cause infection in humans belong to the genus Rhizopus [1].

This was a particularly difficult case with regards to diagnosis and treatment. Early multi-disciplinary team involvement was key in the successful management of this case.

The painless presentation of this lesion and stage IV CKD made the diagnosis of calciphylaxis unlikely [2]. Although biopsy of calciphylaxis lesions is the standard of diagnosis, there is a concern it could lead to new lesions and non-healing ulcers [2]. Therefore, we were reassured in proceeding with a biopsy of the lesion. The patient’s occupation as a farmer and immunosuppressed status was important in considering an infective etiology or mucormycosis as a differential diagnosis for the lesion.

Balancing the immunosuppression dose with the treatment of the mucormycosis was challenging. We had initially lowered the immunosuppression doses to allow treatment of the infection. However, the patients’ kidney function deteriorated as a result. But, the immunosuppression was only titrated up once the infection had been reasonably treated. 

The patient developed severe symptomatic anemia (hemoglobin as low as 6.0 g/dL) as a side effect of the IV Isavuconazole and mycophenolate mofetil. We managed to treat this anemia without giving any blood transfusions to a potential renal transplant recipient by reducing mycophenolate mofetil and switching to oral isavuconazole.

## Conclusions

Absidia corymbifera is a rare cause of mucormycosis. This case highlights the natural history of its transmission (i.e., through the soil in a young immunosuppressed farmer). It is exclusively an opportunistic pathogen and its cutaneous manifestation is a painless, fungating lesion as opposed to a painful lesion seen in calciphylaxis. Early multi-disciplinary team involvement, debridement, and treatment with isavuconazole were key in successfully treating this condition.
